# Establishing sustainable collaborations in global pathology education

**DOI:** 10.3389/fmed.2023.1346780

**Published:** 2023-12-21

**Authors:** Ashley K. Volaric

**Affiliations:** Department of Pathology and Laboratory Medicine, University of Vermont Larner College of Medicine, Burlington, VT, United States

**Keywords:** pathology, laboratory medicine, graduate medical education, low/middle-income countries, global health

## Abstract

Graduate-level pathology education is under-resourced in low/middle-income countries (LMIC) and provides a unique opportunity for building sustainable collaborations. By focusing on a bi-directional educational exchange through graduate medical training in Pathology and Laboratory Medicine (PALM), global collaborations can extend to research and scholarship efforts. There are few PALM-based graduate medical programs in high-income countries (HIC) that offer this type of global educational exchange, and the few that exist have been mitigated by pandemic-related travel restrictions. Nonetheless, re-investing in these types of exchanges will allow for new opportunity in global pathology education and research for the next generation of trainees. Drawing on the author’s own experience in South Africa and Guatemala, five essential elements to establish a sustainable educational collaboration will be discussed: sustained effort and communication between partners of HIC and LMIC, involvement of key stakeholders, educational curriculum involving community engagement and cultural competency, bi-directional exchange between partners, and dedicated time and funding.

## Introduction

Graduate medical education in anatomic and clinical pathology within high-income countries (HIC) like the United States has neglected to engage on the global health front in a systematic manner, with very few formal programs in place (1–3). This is true despite the fact that capacity building in Pathology and Laboratory Medicine (PALM) is desperately needed in low/middle-income countries (LMIC) (4, 5), and pathology trainees have expressed strong interest in this type of engagement (1–3). A sustainable, bi-directional collaboration centered on trainee development and education in PALM services can provide resources and capacity building to LMIC laboratories and medical institutions. This perspective article will explore the tools and elements necessary for building a sustainable pathology educational program, drawing from the author’s own experience in South Africa and Guatemala (6).

**Figure 1:**
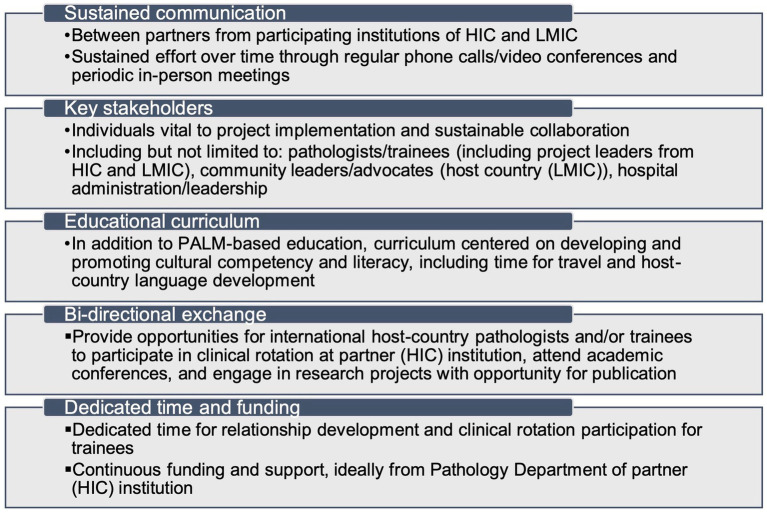
Five essential elements for developing a sustainable global pathology educational program. HIC, high-income country; LMIC, low/middle-income country; PALM, pathology and laboratory medicine.

A successful educational program is one that is sustainable and bi-directional with the host LMIC, meaning there is sustained engagement and proactive learning by the partners with clearly defined academic goals and objectives. Establishing this type of engagement is difficult and time-consuming, but well worth the effort as dividends can be seen in laboratory shared resources, capacity building, education, and research/scholarship projects. In this author’s opinion, there are five essential elements for establishing a sustainable global pathology educational program: sustained effort and communication between partners of HIC and LMIC, involvement of key stakeholders, educational curriculum involving community engagement and cultural competency, bi-directional exchange between partners, and dedicated time and funding (see Figure 1).

### Opening lines of communication

This is perhaps the most important, yet difficult, step. Establishing the communication refers to fostering a sustained open line of communication between the partner institutions of participating HIC and LMIC. This can take the form of regular, scheduled phone calls/video conferences and periodic in-person visits. The partners involved most often will be the pathologists serving as the project leaders from each site, with a respective team of trainees/pathologists, and can involve key stakeholders when appropriate. The importance of this step cannot be overstated, as it ensures trust through establishing a healthy, professional relationship between respective partners. A strong relationship will then allow the details of the project including travel logistics to fall into place more easily.

The author was part of a health education project in the Limpopo Province of South Africa during medical school. The project was implemented over 4 years and involved training community health workers in the initial assessment and management of chronic health disease. The project itself occurred over two summer breaks for 4–5 weeks at a time, but establishing the communication between the institutional partners took well over a year prior. The meetings most often involved planning the project: surveying the community for what kind of education and support was needed, trip logistics, and project implementation with write-up goals and objectives. These meetings, however, can take multiple forms, addressing various aspects of the project itself, but what is important is that there is sustained communication between the partners and time to develop a trusting relationship. Establishing the communication between partners from the respective institutions of HIC and LMIC has been reported as a necessary initial step by multiple studies, often taking at least a year in advance of the rotation itself (7–9).

### Engaging key stakeholders

Key stakeholders are individuals from both partner institutions that are essential to the project development, implementation, and management. These individuals include but are not limited to: pathologists/trainees (including project leaders from HIC and LMIC), community leaders/advocates [host country (LMIC)], and hospital administration/leadership. The incorporation of key stakeholders at appropriate times during project planning can promote sustainability. There is no defined rubric for when to include the key stakeholders during the project planning process, as this should be discussed and agreed upon between the project leaders. However, it is likely that for a pathology clinical rotation at an international site, the key stakeholders will attend only a subset of meetings. It is important that when invited to a planning meeting, the key stakeholders (often hospital administrators/leadership or community leaders/advocates) are brought into the conversation in a respectful manner and personally asked for their input and opinion on the invited topic. This type of engagement will strengthen the established trust and communication between the sites.

The key stakeholders for the health education project in South Africa included leadership from the Ministry of Health, who also served as community leaders and advocates. For the pathology clinical rotation in Guatemala (6), the key stakeholders included hospital administration/leadership and a community leader/advocate, who also served as the on-site project coordinator. For both projects, the key stakeholders were invited to specific meetings where their knowledge and expertise were called upon, and these meetings were often by phone or video conference. Follow-up discussions occurred during in-person site visits.

### Formalizing the educational curriculum

Ideally, the PALM clinical rotation at the host institution should last at least 4 weeks, to allow for a culturally-rich educational experience. The educational curriculum, in addition to the PALM-based education, should include cultural competency and language, while allowing time for travel. The cultural competency component of the educational curriculum for the Guatemala project included preparation prior to the rotation: 10 h of online Spanish language lessons and required cultural reading. During the rotation, the first week consisted solely of in-person, immersive Spanish language school with dedicated time in the afternoons for activities hosted by the school and on the weekend for travel to heritage sites. The clinical rotation (3 weeks) allowed and encouraged travel on the weekend. During the week, the trainees met with the in-country project coordinator to discuss the required reading and topics pertinent to the Mayan culture and health/medicine of Guatemala. Further, the trainees stayed at family homestays and were fully immersed in the Spanish language. This type of deep cultural immersion embedded into the educational curriculum allows for a more complete understanding of how PALM intersects with culture on the global front.

The PALM-based portion of the educational curriculum should be developed in collaboration with the host pathologist (project leader) of the LMIC. Depending on the PALM focus (anatomic versus clinical pathology), the day can be subdivided by independent case review and structured case sign-out. At the conclusion of the rotation, a formal mechanism for feedback should be in place to allow the project leaders to continually improve and refine the experience. Feedback should be freely given by participating trainees, as well as solicited at regular intervals from the key stakeholders. Project leaders should also maintain established communication and solicit feedback from each other. This educational framework has been employed and promoted by other global pathology rotations (7) in addition to the author’s own work in Guatemala (6). In addition, it is important to work closely with the Accreditation Council for Graduate Medical Education (ACGME)/GME to codify the curriculum as it pertains to postgraduate medical training. The stipulations and restraints of the ACGME/GME should serve to strengthen and not hinder the educational experience for the pathology trainees.

### Establishing bi-directional exchange

A bi-directional exchange refers to having pathology trainees of the partner institution complete the clinical rotation in the host LMIC and providing opportunity for the host pathologists and/or trainees to complete a clinical rotation or attend a major academic conference in the partner HIC. The latter exchange should be carefully coordinated in concert with ACGME/GME requirements, which can limit the number of learners allowed from the host LMIC at a given time. Further, standards of patient confidentiality and safety should be upheld. Restraints in this regard can be circumnavigated by promoting the rotation as a directed student observership. This allows for a bi-directional educational experience where both partners are engaged and actively learning, which is crucial for ensuring project sustainability.

This approach has been employed in the author’s work in Guatemala (6), where the host pathologist was supported by the partner institution to attend a major academic conference and visit with the Pathology Department. A study detailing a pathology rotation established in Trinidad & Tobago emphasized the need for a bi-directional exchange, with the ultimate goal of providing an equitable clinical rotation at the partner institution (7). Other projects have highlighted a unique educational exchange where students of the partner LMIC participate in a tailored clinical rotation at the HIC institution (10). The bi-directional exchange can also take the form of a recurring telepathology educational and clinical conference, both employed in the author’s work in Guatemala (6) and other institutions (11). Finally, the bi-directional exchange can be utilized to share quality improvement and other capacity-building strategies between the partners. Regardless of the form, a bi-directional educational exchange is essential to ensuring project sustainability and nurturing shared interest and engagement.

### Securing dedicated time and funding

Dedicated time and funding are necessary for a successful, sustainable global pathology educational collaboration. Trainees who are participating in the rotation need be allowed the appropriate time away from clinical duties at their home institution, without retribution or withholding of salary. This requires active engagement and agreement by the ACGME/GME office as well as the Pathology Department. In addition, dedicated off-service time is necessary for the project leader to engage in sustained communication with the host-country partners for the purpose of project planning, implementation, and maintenance.

Dedicated funding is crucial for the success of any collaborative project, but in the global health arena, goes further in promoting project sustainability. Ideally, a modest portion of funds can be provided annually by the partner Pathology Department of the HIC. These funds can help cover trainee travel cost and provide rudimentary support for the host pathologist. In addition, equipment and books from the partner institution can be donated. Another approach can be to establish a research program and use the extramural funding for resource and project support (12). Multiple pathology organizations also offer trainee grants and funding for global health education and research efforts (1).

## Conclusion

Establishing a truly sustainable educational collaboration in global pathology is difficult but well worth the effort. There are many ways to create such a collaboration. However, in this author’s opinion, there are five essential elements for project sustainability: sustained effort and communication between partners of HIC and LMIC, involvement of key stakeholders, educational curriculum involving community engagement and cultural competency, bi-directional exchange between partners, and dedicated time and funding. Sustainable educational collaborations with LMIC partners can pave the way for remarkable pathology education and new avenues for translational research. Such efforts are tremendously needed and garners learning for all those involved.

## Data availability statement

The raw data supporting the conclusions of this article will be made available by the authors, without undue reservation.

## Author contributions

AV: Conceptualization, Writing – original draft, Writing – review & editing.
